# Tendinopathy: sex bias starts from the preclinical development of tendon treatments. A systematic review

**DOI:** 10.1186/s13293-022-00453-z

**Published:** 2022-07-30

**Authors:** Camilla Mondini Trissino da Lodi, Manuela Salerno, Giulia Merli, Pieter Brama, Florien Jenner, Giuseppe Filardo

**Affiliations:** 1 Service of Orthopaedics and Traumatology, Department of Surgery, EOC, 6900 Lugano, Switzerland; 2 Applied and Translational Research Center, IRCCS Istituto Ortopedico Rizzoli, 40136 Bologna, Italy; 3 Section Veterinary Clinical Sciences, School of Veterinary Medicine, University College Dublin, 4 Dublin, Ireland; 4 Equine Surgery Unit, University Equine Hospital, Department of Companion Animals and Horses, University of Veterinary Medicine Vienna, Veterinaerplatz, 1210 Vienna, Austria; 5 Faculty of Biomedical Sciences, Università Della Svizzera Italiana, 6900 Lugano, Switzerland

## Abstract

Tendinopathies are common overuse disorders that arise both in athletes and the general population. Available tendon treatments are used both for women and men without distinction. However, the existence of a sex-based difference in tendon biology is widely demonstrated. Since basic research represents the foundation for treatment development, an equal female–male representation should be pursued in preclinical studies. This systematic review quantified the current evidence by analyzing 150 studies on 8231 animals. Preclinical studies largely neglected the importance of sex, none analyzed sex-based differences, and only 4% of the studies reported disaggregated data suitable for the analysis of treatment results in males and females. There is an alarming female under-representation, in particular in the field of injective therapies. Despite the growing awareness on the importance of investigating treatments in both males and females, the investigated field proved resistant from properly designing studies including both sexes, and the lack of sex-representation remains critical.

**Supplementary Information:**

The online version contains supplementary material available at 10.1186/s13293-022-00453-z.

## Highlights

- Tendinopathy has sex-specific features, with sex hormones affecting tendon metabolism, structure, biomechanical properties, and injury risk.
- The preclinical research on tendinopathy treatments still neglects sex-based differences, leading to translation of male data to females which may affect clinical effectiveness in women.
- None of the reviewed studies looked at differences between sexes, and only 4% of the studies reported disaggregated data. Besides, female animals are under-represented.
- The lack of sex-representation in tendinopathy research remains critical.

**Supplementary Information:**

The online version contains supplementary material available at 10.1186/s13293-022-00453-z.

## Introduction

Tendinopathies are common overuse disorders that arise both in athletes and the general population [1]. Chronic tendon degeneration results in transient or persisting pain and may progress to reduced function up to partial or total tendon rupture [2–5]. As a consequence, these pathologies represent a huge socio-economic burden in terms of impact on patients’ sports activity and everyday life, loss of working productivity and need of indemnity for disease [1]. To address tendinopathy, while a few recalcitrant cases require surgical treatment, most of the affected patients are managed by conservative approaches [1, 4, 6]. Among these, exercise-based strategies—alone or associated with extracorporeal shockwave therapy (ESWT)—have been the most described treatments reaching a broad support in clinical practice. Moreover, intralesional injection of products such as platelet-rich plasma (PRP) or other biological substances is also emerging as promising minimally invasive therapeutic options [7].

Tendon treatments are used both for women and men without distinction. Available literature, however, widely demonstrated the existence of a sex-based difference in tendon biology. More so, men and women are characterized by deeply different metabolisms, hormonal balances, histological, and even anatomical differences which require and warrant scientific differentiation and investigation. This could improve the understanding of biological pathways, which have clinically relevant effects like the well-documented higher prevalence of tendon and or ligament injuries in female athletes than in males [8–11]. This is even more important considering the increasing number of active women. Unfortunately, various examples in the scientific literature show a general tendency of translating male data into females, leading to the so-called gender bias, defined as the absence of female scientific data due to cultural influences that overlook women [12]. However, biological differences should not be neglected since they could lead to a different response to treatments. This concept has been stressed by several international organizations, since the Revitalization act of 1993 started requiring female inclusion in National Institutes of Health (NIH)-founded clinical research [13]. Recently, applicants for the NIH are expected to explain how they will account for Sex as a Biological Variable and to provide a justification for single-sex studies [14]. Since basic research represents the foundation for treatment development, an equal female–male representation should not pertain only to clinical trials, but also to preclinical studies.

The aim of this study was to quantitatively analyze the preclinical literature, to identify evidence on sex-based differences in the studies performed to assess conservative treatments for tendinopathy.

## Methods

### Literature research and selection criteria

A systematic review of the literature was performed on June 1, 2021 and was conducted in the bibliographic databases PubMed, Web of Science, and Wiley Cochrane Library, with no time limitation and without any filter, using the following string: (exercise OR “extracorporeal shockwave” OR inject*) AND (tendinopat* OR tendon OR tendinitis OR tendinosis) AND ((mouse) OR (rat) OR (rodent) OR (rabbit) OR (lapin) OR (dog) OR (canine) OR (sheep) OR (goat) OR (horse) OR (pig) OR (swine) OR (bovine)). According to the guidelines for Preferred Reporting Items for Systematic Reviews and Meta-analysis (PRISMA) [15], the screening process and analysis were separately conducted by two authors (CM, MS), using inclusion criteria: research articles on animal models of tendinopathies, treated with exercise, ESWT, or injection therapies, and written in the English language, without time limitation. Studies in other languages, in vitro or clinical studies, and literature reviews and meta-analyses were excluded.

### Data extraction

**Fig. 1:**
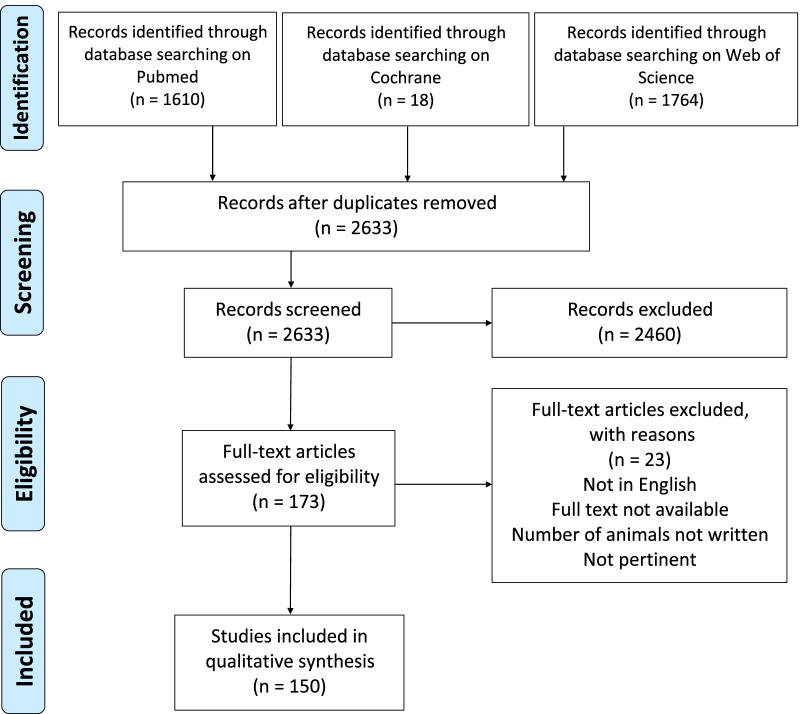
PRISMA flowchart of the study selection process

The selected studies were first screened by title and abstract. In the second step, the studies that met the inclusion criteria were further screened for full-text eligibility according to the previously described criteria. In case of disagreement between the two reviewers, discrepancies were resolved by discussion and consensus with a third author (GM). An electronic table for data extraction was created prior to the study using Excel (Microsoft). The relevant data were then extracted: title, first author, year of publication, journal, animal model, tendinopathy induction, involved tendon, type of treatment, total number of animals, number of males and females, outcomes disaggregated by sex or not, and discussion of gender-related limitations. The studies were grouped in three main groups based on the treatment: exercise, ESWT, and injective treatment. If a study compared two distinct managements, they were counted individually for each category. The gender reporting bias trend was evaluated over time based on 5-year periods. A PRISMA flowchart of the screening process is shown in Fig. 1.

### Statistical analysis

Frequencies and percentages were used to report types of studies, presence of sex reporting, and the sex of the used animals. The proportions of the studies and the animals used in the studies of each group under observation were evaluated every 5 years from 1996 to 2020. The 95% confidence interval was evaluated according to the continuity-corrected Wilson interval (Newcombe, 1998a). The pooled expected value of each group under observation was evaluated using the Mantel–Haenszel methods (Mantel 1959, Greenland 1985). With no heterogeneity, the estimation of the expected value and its 95% confidence interval were based on fixed effect analysis of variance. Statistical heterogeneity was evaluated by the I-square statistic and Cochran’s Q. The comparison among the groups was based on the Z-test with Bonferroni correction for multiple comparisons.

### Assessment of risk of bias and quality of evidence

Due to study aim, assessment of risk of bias and quality of evidence were not required. Animals’ type and sex were not linked to the other aspects of the quality of the studies.

### Institutional board review and funding source

An institutional review board endorsement was not required because all data were extracted from previously published studies. No external funding was received for the initiation or completion of this study.

## Results

### Selection of the studies

A total of 3392 items were identified after the three-database search, 759 of which were duplicates. Of the 2633 records, 2143 were excluded by title, and 317 by abstract. Therefore, a total of 173 studies were eligible for the full-text analysis. After the last step, further 23 studies did not fulfill the inclusion criteria because treatments or lesion models were not pertinent to the study topic, or the number of animals was unclear. Finally, 150 studies, published between 1986 and 2021, were fully eligible for the analysis (Additional file 1).

### Study characteristics

**Fig. 2:**
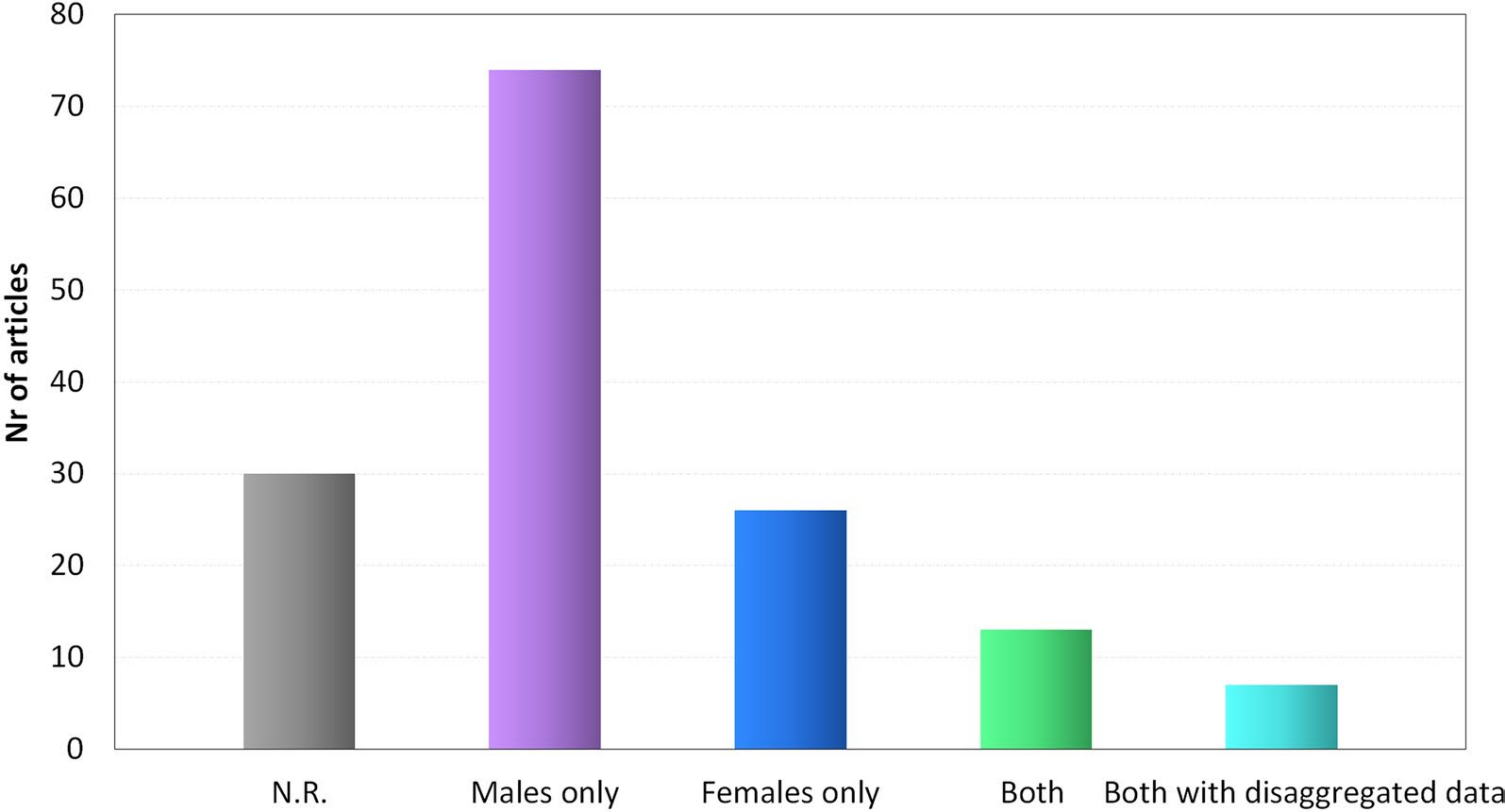
Sex representation in the analyzed studies. Total number of studies including only males, only females, or both sexes. N.R. = not reported

A total of 8231 animals were enrolled in the 150 included papers: 30 studies (20%) did not specify animal sex (1788 animals, 22%), whereas 120 stated it (6443 animals, 78%). Among these, 20 (17%) with 386 animals included both males (219 animals, 57%) and females (167 animals, 43%), and out of these 7 (35%, corresponding to 5% of the total number of literature studies analyzed) disaggregated data by sex; 74 studies (50%) used males only (4351 animals), whereas 26 (17%) used females only (1706 animals) (Fig. 2).

Among the 20 studies including animals of both sexes, 13 did not analyze data by sex (one reported a general indication on sex-related results without indicating specific data), 1 analyzed two different types of outcomes for males and females, and 6 studies disaggregated results by single animals (Fig. 2). One study considered the inclusion of only or mostly animals of one sex as a limitation of the study, stating that female hormonal fluctuations may confound results and thus focusing only on males. The studies included both small and large animals (Table 1) and different tendons were involved (Table 2). Both spontaneous (22 studies, 15%) and induced tendinopathies were treated (128 studies, 85%). In the latter case, different methods were used to induce the lesions, including surgery (83 studies, 65%), injection of chemical substances (38 studies, 30%), or mechanical overloading (6 studies, 4%). In 1 study (1%), lesions were induced by a combination of mechanical and chemical methods.

**Table 1:** Animal models used in the studies

Animals	Nr of studies	Males	Neutered males	Females	Neutered females
Large animals	39	206 (48%)	146 (71%)	222 (52%)	38 (17%)
Horses	31	124 (60%)	97 (78%)	82 (40%)	0 (0%)
Dogs	4	78 (58%)	49 (63%)	57 (42%)	38 (67%)
Sheep	4	0 (0%)	0 (0%)	83 (100%)	0 (0%)
Small animals	111	4364 (73%)	0 (0%)	1651 (27%)	0 (0%)
Rabbits	27	391 (77%)	0 (0%)	116 (23%)	0 (0%)
Rats	79	3285 (70%)	0 (0%)	1395 (30%)	0 (0%)
Mice	5	688 (83%)	0 (0%)	140 (17%)	0 (0%)

Number of enrolled animals and, in brackets, the corresponding percentages. Animals included in the studies that did not specify the sex were not reported

**Table 2:** Tendons analyzed in the studies

Tendons	Nr of studies	Animal models	Males	Females
Achilles	84	Rat (70%), rabbit (20%), mouse (6%), sheep (4%)	3409 (72%)	1314 (28%)
Superficial digital flexor	30	Horse (97%), rabbit (3%)	139 (67%)	68 (33%)
Patellar	16	Rat (88%), rabbit (6%), mouse (6%)	504 (68%)	236 (32%)
Rotator cuff	17	Rat (52%), dog (24%), rabbit (24%)	525 (75%)	177 (25%)
Deep digital flexor	8	Rabbit (50%), horse (38%), rat (12%)	83 (55%)	67 (45%)
Peroneus	1	Horse (100%)	18 (72%)	7 (28%)

Used animal models and, in brackets, the percentage of studies in which they have been used. Total number of enrolled animals by sex and, in brackets, the corresponding percentages

### Sex representation in the preclinical literature

Among the 8231 enrolled animals, for 1788 (22%) sex was not described, while for 6443 (78%) sex was reported. Out of these, 4570 were males (71%), whereas 1873 were females (29%). However, 118 males and 93 females were described together in the same study outcome without reporting sex-based results. With regard to sex representation in terms of results data availability, 4351 males (71%) and 1736 females (29%) were studied separately in studies involving only one sex. Out of the studies reporting both sexes and sex-related results, one study analyzed different outcomes for each sex, while 6 studies described the results individually for each animal (4%), but without analyzing sex-related differences.

### Sex representation and animal models/treatments

**Fig. 3:**
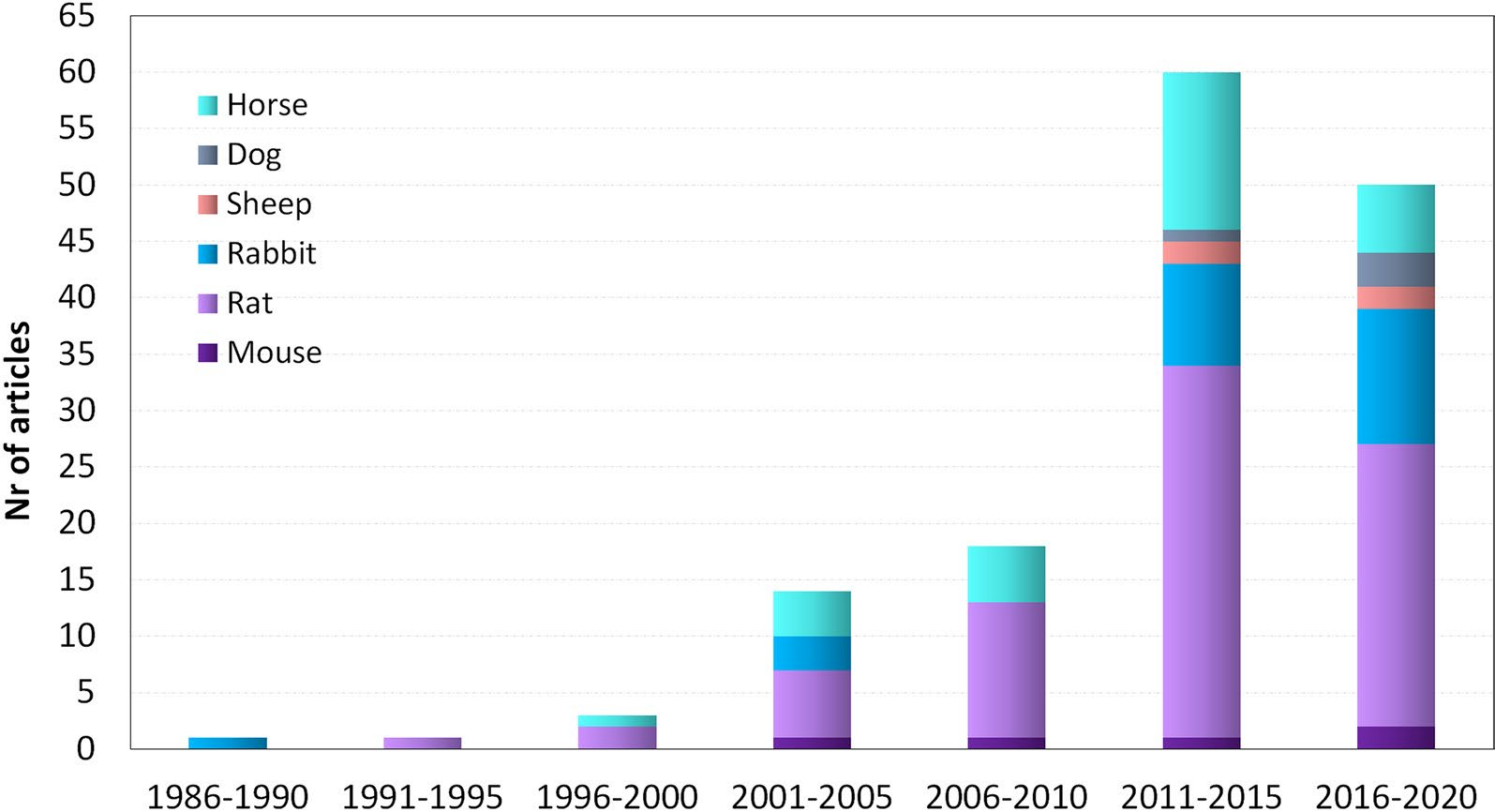
Changes in animal models over time. Analysis of the different animal models included in the retrieved studies over the years

**Fig. 4:**
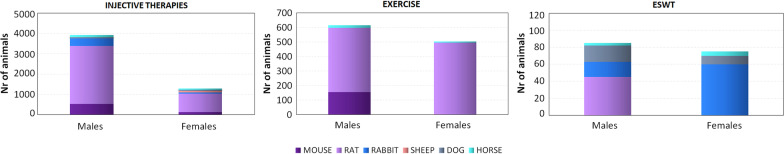
Treatments. Number of males and females in the three main treatment categories divided per animal model

Large heterogeneity has been observed both in males and females concerning the animal model used for the evaluation of different treatments. The type of animal model did not change over time (Fig. 3). Overall, rats were the most represented model (more than 50% of the studies over the years). Disaggregated male and female data were reported in a few studies on dogs (2 out of 4, 50%) and horses (4 out of 31, 13%) (Fig. 4). In these two large-animal models, a large percentage of cases was represented by neutered animals (44%). In particular, 71% of large male animals were castrated and 18% of large female animals were neutered (2% of all female animals, including large and small models, were neutered) (Table 1).

Injective treatments were the most studied (126 studies, 84%), followed by exercise (21 studies, 14%), and by ESWT (7 studies, 5%). In 4 of these studies, exercise and injective treatments were used in combination. Among the injective treatments, used alone or in combination, the most used were blood derivatives (52 studies, 42%), followed by the injection of cells obtained from various sources (50 studies, 39%), hyaluronic acid (HA) (12 studies, 10%), and corticosteroids (9 studies, 7%), while 19 studies investigated other substances (15%).

Literature data were further analyzed to determine sex representation and sex-related results in the three main treatment categories (Fig. 4). Of the 126 studies researching injective therapies and in which research subject sex was reported (including 5215 animals in total), subject level (single animal) reporting was done in 1 study in dogs and 3 studies in horses, totaling 26 males and 20 females, 0.5% and 0.4%, respectively. Of the 99 studies that reported animals’ sex, 61 (62%) studies reported only on male and 19 (19%) only on female animals.

Among the 21 studies focused on exercise, 18 investigated large and 3 small animal models, respectively. 11 (61%) studies reported only on male and 5 (28%) only on female animals. Considering the overall 1118 animals studied to develop exercise tendon treatments, one study on rat with 36 females (3%) and 52 males (5%) disaggregated the results of the study for animal sex at a subject level.

Among the 7 studies focusing on ESWT, 5 investigated large and 2 small animal models, respectively. Two studies (33%) reported only on male and 2 (33%) only on female animals. One study on dogs and 1 study on horses reported the results of the study separately at a subject level, for a total of 15 females and 22 males, 9% and 14%, respectively, of the overall 160 animals studied to develop ESWT.

### Sex-representation trend over time

**Fig. 5:**
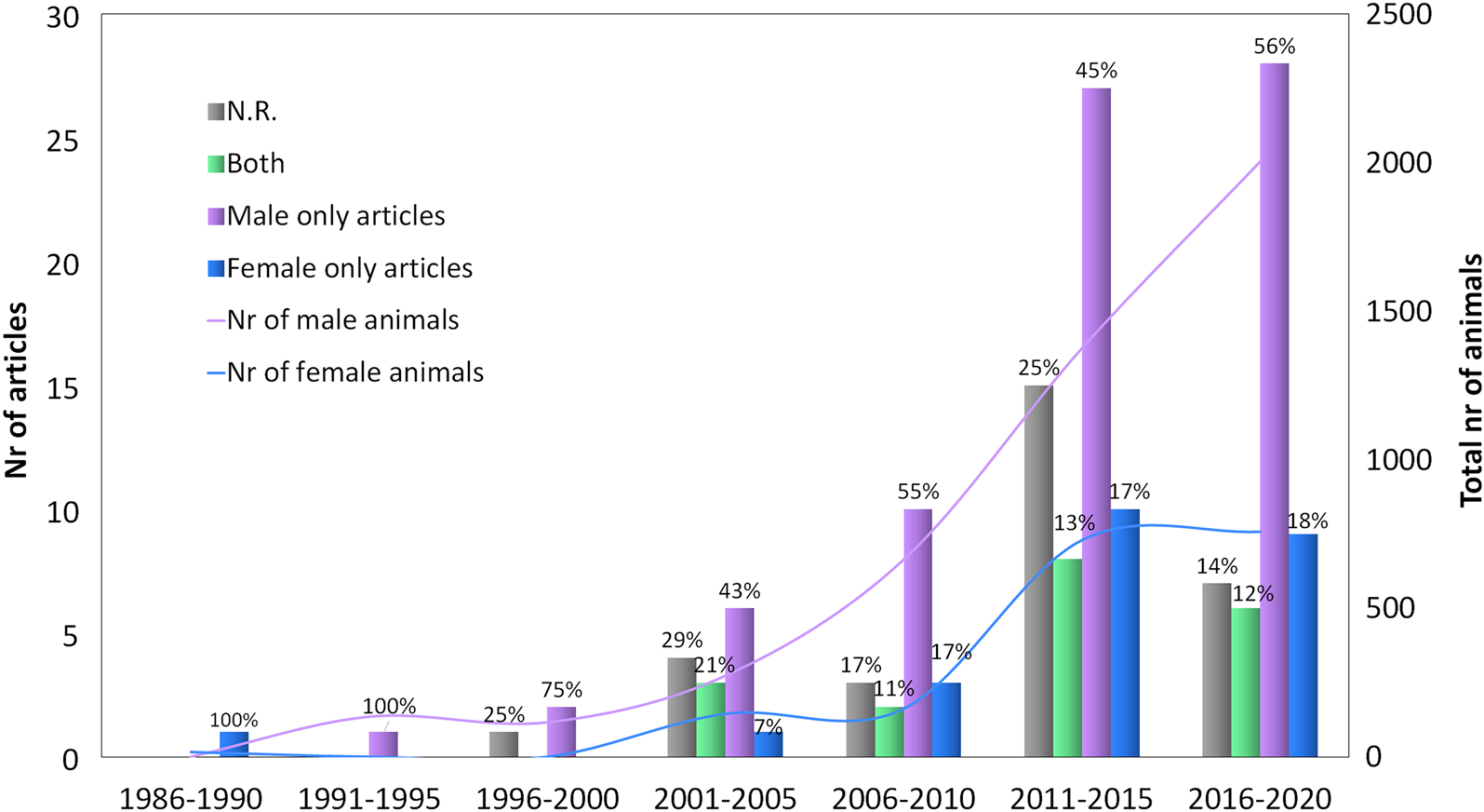
Trend of sex-representation. Sex-representation in the published studies and total number of male/female animals over the years. The percentages are calculated with respect to the total number of studies published on each time interval. N.R. = not reported

From the first publication up to 2020, a statistically higher number of male animals were documented (*P* < 0.001). In the last 10 years, the number of male studies increased significantly more than the number of female studies and of studies including both sexes (*P* < 0.05) (Fig. 5).

## Discussion

The main finding of this study is that preclinical studies performed to assess treatments for tendinopathies currently used in clinical practice largely neglected the importance of sex. None of the published studies analyzed sex-based differences, and only 4% of the studies reported disaggregated data suitable for the analysis of treatment results based on sex. Beside the low number of studies including both sexes, there is an alarming female under-representation in the study subjects, in particular for injective therapies. Despite the growing awareness of the importance of investigating treatments in both males and females, the field proved resistant from properly designing studies, and the lack of sex-representation remains critical.

The notion of the importance of sex and gender is not new to the scientific literature, which defines sex as biological and physiological features characterizing male and female individuals, while gender refers to socially constructed roles, behaviors and identities of female, male, and gender-diverse people [16, 17]. Since the NIH Revitalisation Act came out in 1993, several international organizations encouraged a broader female inclusion in both preclinical and clinical studies [13]. Between 2005 and 2008, the Gender Basic Project in Europe supported scientific studies focusing on sex and gender and on the importance of both sexes’ outcomes. Few years later, the European Association of Science Editors (EASE) established a Gender Policy Committee with the aim to develop a set of guidelines for reporting of Sex and Gender Equity in Research (SAGER) [17]. More recently, in 2012, the Canadian Institute of Health, as well as the German Society of Epidemiology, mandated a justification for any study if only one sex was considered [18, 19]. In 2016, NIH requested all applicants to provide a justification in case of one-sex studies [14]. Considering the several authoritative and pressive guidelines requiring the field to address this key issue, one may expect a literature evolution toward a more balanced sex representation. Surprisingly, this is not the case, and the literature is showing a significant worsening trend. In fact, despite the 2016 NIH request for looking at Sex as a Biological Variable, this systematic review on one of the most common orthopedic pathologies, tendinopathy, showed that the number of male specific studies increased more than the female focused studies, while the percentage of studies including both sexes even decreased.

This is sadly not a surprising result. Bryant et al. in 2018 analyzed four orthopedic journals and found that only 13% of the studies disaggregated data by sex [20]. While this issue is less explored in the orthopedic field, these results echo many non-orthopedic studies denouncing the unrecognized female relevance in the preclinical medical research. Over a decade, more than 79% of animal studies published in the *Pain* journal were on male animals, while only 4% were explicitly focused on testing sex differences [17]. In cardiovascular research, Ramirez et al. found that only 13% and 15% of the studies included females and both sexes, respectively [21]. Low percentages of female inclusion and data disaggregation can also be found in a long list of other disciplines, including basic science, dermatology, neurosciences, pharmacology, otolaryngology, etc. [17, 22–26]. As emerged from this systematic analysis of the literature, female neglection reaches an alarming level in the research efforts to develop solutions to address tendinopathies, a common and debilitating clinical challenge. Beside the marginal effort on including both females and males in the same experiments, no study recognized the lack of comparative sex-based analysis as a limitation, and 20% did not even considered it useful to report the sex of the studied animals. Only 13% of the studies included both males and females and, among these studies, only 6 disaggregated results by sex, representing only 4% of the total studies, which include an even lower number of animals (2%). In these studies, moreover, the results have been disaggregated due to the description at a subject level but without the analysis on the influence of sex, thus reflecting the fact that not one of these authors consciously looked out for sex-based outcomes.

Men and women are characterized by differences resulting from millions of years of evolution and therefore warrant scientific differentiation and investigation. This applies both to men and women. Men should be better studied with more representative models, since 71% of the large male animals documented in the literature were castrated, which could affect the metabolism and physiological treatment response, hindering the translatability of the study findings in humans. In women, estrogen receptors (ER) have been localized in both ligaments and tendons, and an increase of estrogen concentration has been linked to a decreased collagen synthesis and a lower tendon stiffness [8, 27, 28]. Also, it has been hypothesized that the hormonal fluctuations related to the menstrual cycle and menopause may influence the incidence of musculoskeletal injuries, with an increasing risk in the ovulatory phase and in menopausal women, respectively [29–32]. Based on these premises, some attempts of investigating sex-based differences in terms of tendon biology have been performed in animal models. In rats, it was demonstrated that ageing and more significantly estrogen deficiency negatively affect tendon metabolism and healing rate. A decrease in fibronectin and elastin, an increase in vascular endothelial growth factor and Metalloproteinase-13, and a low healing rate of microwounds have been found in tenocytes of estrogen-deficient rats when compared to young and old groups [33]. In mice, male and female tendons differ for extracellular matrix proteins and proteoglycans composition, mechanical properties, gene expression, protein composition, resistance to mechanical stress, and response to therapy [34–36]. The combined effects of estrogen and mechanical loading may alter the mRNA expression for extracellular matrix components exclusive of females, supporting the higher injury risk in females [37], and a correlation has been suggested between the expression of estrogen receptor-beta and mechanical stress in rat tendinopathy [36]. Despite this evidence, few studies are currently available on this topic and the results are not conclusive, which underlines the importance of further exploring sex-related differences in both etiopathogenetic mechanisms and treatment development.

Given the sex-specific incidence of tendinopathy, with sex hormones affecting tendon metabolism, structure, biomechanical properties and injury risk, and the interplay with age-related tendon modifications, sex differences should be studied across the entire lifespan to gain insight into disease pathogenesis and identify treatment targets for different sexes and times of life. The incidence of tendon injuries increases dramatically with age-related changes in tendon structure, composition, mechanical function, and injury risk which appears to be sex-dependent, with the incidence of tendinopathy rising following menopause [38, 39]. All these aspects are largely overlooked in the preclinical literature. For example, no animals in physiologic menopause were studied, and only 2% of the female animals were neutered. Commonly used animal model species do not naturally undergo menopause with its associated decrease in estrogen levels and ER expression. Ageing rodents fail to consistently replicate the low estrogen concentrations characteristic of human menopause [40, 41]. Traditionally, methods to induce menopause in animals have focused on ovariectomy, which yields a substantially different hormone profile, with a sudden loss of all ovarian steroids rather than continued release of androgens and low levels of other steroids, as well as an altered hypothalamic–pituitary–gonadal axis compared to post-menopausal women [41]. The common use of sexually immature animals, which distorts the effect of ovariectomy, lends further emphasis to the necessity for fit-for-purpose animal models in general and in specific for research into sex- and age-specific pathogenetic and reparative mechanisms [41, 42].

Neglecting a sex-based analysis in preclinical studies might lead to a great bias, with a relevant impact on the translational research. The improper sex-representation in the preclinical research of tendinopathy has been often justified by the fear of a more complex model due to hormonal fluctuations. This led to the overall lower inclusions of female animals, mainly limited to the clinical veterinarian studies in dogs and horses, while experimental studies overly draw their attention to male small animal models. In rodents, the exclusion of female seems to be systematic, even though no justification for this selection bias is provided by scientific evidence [43–46]. Indeed, there is the improper belief that females are subjected to a greater variability, due to the confounding effect of the estrus cycle, making them unstable and unsuitable for their use as preclinical models. This myth has been questioned several times. Prendergast et al. in 2014 analyzed 293 studies on biomedical research and were able to prove not only that females do not express more variability, but also that male mice could be even more variable, due for example to the group-housing conditions that can lead to fight and consequent hormonal pathways activation [43]. In 2016 another meta-analysis of neuroscience studies confirmed that female rats exhibited the same, or even less, variability than males [47]. This was true for behavioral, electrophysiological, neurochemical, and histological measures. Thus, the authors concluded that power analyses based on variance in male measures are sufficient to yield accurate numbers for females as well when designing experiments to include both male and female rats. To challenge the assumption of inherently greater female variability, Itoh et al. analyzed a large microarray data set measuring gene expression in various tissues of both mice and humans, comprising the analysis of more than 5 million probes [44]. On average, male gene expression was slightly more variable than that of females, reaching again the same conclusion of no evidence for greater variability in females than in males. Thus, the scientific evidence does not justify male selection in preclinical biomedical research.

The development of treatments requires the study of both sexes. Ignoring sex-disaggregation and female specific effects in a preclinical phase can lead, in the best-case scenario, to missing the opportunity of investigating such effects in clinical studies. The consequences might be not trivial, as for the adverse events in women, for instance, with the case of Zolpidem in 1992. This drug was approved and commercialized with the same dosage for both sexes and, 10 years later, it was halved by FDA in women due to sex-specific severe side effects [48, 49]. Between 1997 and 2001, ten drugs have been withdrawn from the US market and eight of them were more harmful to women [50]. While the importance of sex-related differences goes across all fields in biomedical research, the implementation of studies with a proper study design is severely lagging, as vividly portrayed by the current meta-analysis on one of the most common orthopedic diseases. At best, the importance of comparing results of both sexes has been underestimated. Experimental models used to develop the main conservative treatments for tendinopathy focused their attention of only one sex, most commonly on male animals. This is surprisingly even more true for the newest treatments introduced in the clinical practice, the injective approaches. In fact, innovative blood derivatives and cell-based approaches have been tested and implemented in clinical practice without considering potential sex-related risks and possible sex-related differences in terms of healing potential, even though orthobiological solutions could be particularly influenced by the biological differences characterizing males and females [7, 51, 52].

This literature analysis was focused on a common pathology, tendinopathy. Still, a limitation is that different types of tendinopathies have been grouped together for the purpose of this study. Thus, different tendons and treatments may present more or less bias in terms of depth of investigation. However, this goes beyond the purpose of this study, which was focused on a broader concept of sex-representativeness in the overall research field on the development of tendon treatments. To this aim, this study encompassed the entire preclinical literature on conservative tendon treatments, reporting on 150 studies and 8231 animals, and unquestionably identified a critical field limitation due to a severe sex bias, even though it should be underlined that a minority of them on horses and dogs were not intended to develop new treatments, being per se clinical veterinary studies, therefore being outside the translational preclinical context. The enrollment of both sexes should be pursued in future studies. Females do not have a substantial increase in outcome variance, irrespective of the cycle state [27, 45, 53]. Also, if a specific hormonal influence is suspected to be a further complex study variable, the enrollment of both sexes is even more strongly recommended, to better understand how to properly address tendinopathies in women.

Proper gender-balanced studies are needed. Still, this may prove difficult. Preclinical research presents a delicate balance of several aspects which weigh in defining the study design. Having both males and females in every experiment faces impediments both on ethics (increased animal numbers vs the need for “reduction”) and finances (increased budgets required due to higher number of animals and increase in purchase, housing costs, etc.), as well as practical limitations to properly implement environmental/phenotypic study setting (for instance male goats together will be much more active fighting than a female group in normal housing conditions) [54, 55]. Finding the proper balance would require guidelines on how to properly power studies detecting a sex difference based on the specific animal model and study target, while also giving guidance on the most suitable settings to derive sex-based results, especially in terms of preclinical data to translate effective musculoskeletal treatments in women.

Women are more at risk of sustaining tendon and ligament injuries [10, 56, 57], and their growing sport participation urges a decisive change of direction in preclinical tendon studies to provide specific data to develop more suitable treatments for both men and women affected by tendinopathies.

## Perspectives and significance

Tendon metabolism, structure, biomechanics, and injury risk are strongly influenced by sex hormones. In this study, we highlighted, through the analysis of a large number of preclinical data, that female sex is neglected in the research focused on the development of therapies for tendinopathy. We proved the female under-representation both in terms of animals’ number and of sex-related results disaggregation. This lack of data confirms the existence of a great sex bias also in this field, which is alarming given that preclinical research is the foundation of human clinical trials, and tendinopathy represents one of the most common and debilitating musculoskeletal disorders affecting healthy active people, more often women. We hope that sharing these findings could stimulate a significant change of direction in the in vivo preclinical efforts to develop new treatments, toward the achievement of a more equal gender-based research, with important consequences for women’s health.

## Conclusions

This study demonstrated an important sex bias in the orthopedic field of preclinical research for the development of tendinopathy treatments. None of the published studies analyzed sex-based differences, and only 4% of the studies reported disaggregated data suitable for the analysis of treatment results in males and females. There is an alarming female under-representation in the studied animals, in particular for the field of the new injective therapies. Despite the growing awareness on the importance of investigating treatments in both males and females, the field showed a worsening trend with an increasing number of male-centered studies and fewer studies comparing treatment results in both males and females. The lack of sex-representation in tendinopathy research remains critical.

## Supplementary Information

**Additional file 1.** Supplementary Material. Studies included in the Systematic Review.

## Data Availability

Not applicable.
