# Supplementary material for: Tendinopathy: sex bias starts from the preclinical development of tendon treatments. A systematic review
Source: Biol Sex Differ. 2022 Jul 30;13:44. doi: 10.1186/s13293-022-00453-z (PMC9338527; doi:10.1186/s13293-022-00453-z)
Supplement: Supplementary file 1 — Additional file 1. Supplementary Material. Studies included in the Systematic Review. [file 13293_2022_453_MOESM1_ESM.docx]

**Supplementary Material: Articles included in the Systematic Review**

1. Ahrberg AB, Horstmeier C, Berner D, Brehm W, Gittel C, Hillmann A, et al. Effects of mesenchymal stromal cells versus serum on tendon healing in a controlled experimental trial in an equine model. *BMC Musculoskelet Disord* 2018;**19**:230.

2. Allahverdi A, Sharifi D, Takhtfooladi MA, Hesaraki S, Khansari M, Dorbeh SS. Evaluation of low-level laser therapy, platelet-rich plasma, and their combination on the healing of Achilles tendon in rabbits. *Lasers Med Sci* 2015;**30**:1305–13.

3. Alves ALG, Rodrigues MAM, Aguiar AJA, Thomassian A, Nicoletti JLM, Hussni CA, et al. Effects of beta-aminopropionitrile fumarate and exercise on equine tendon healing: Gross and histological aspects. *J Equine Vet Sci* 2001;**21**:335–40.

4. Argüelles D, Carmona JU, Climent F, Muñoz E, Prades M. Autologous platelet concentrates as a treatment for musculoskeletal lesions in five horses. *Vet Rec* 2008;**162**:208–11.

5. Arslan I, Yucel I, Ozturk TB, Karahan N, Orak MM, Midi A. The effects of corticosteroid injection in the healthy and damaged achilles tendon model: histopathological and biomechanical experimental study in rats. *Turk Patoloji Derg* 2020;**36**:39-47.

6. Aspenberg P, Virchenko O. Platelet concentrate injection improves Achilles tendon repair in rats. *Acta Orthop Scand* 2004;**75**:93–9.

7. Azad-Tirgan M, Sarrafzadeh-Rezaei F, Malekinejad H, Hobbenaghi R, Heshmatian B. Evaluation of tendon healing using fibroblast like synoviocytes in rabbits: A biomechanical study. *Vet Res Forum* 2016;**7**:21–6.

8. Balesdent Barreira AP, Garcia Alves AL, Saito ME, Amorim RL, Kohayagawa A, Menarim BC, et al. Autologous Implant of Bone Marrow Mononuclear Cells as Treatment of Induced Equine Tendinitis. *Intern J Appl Res Vet Med* 2008;**6**:46–54.

9. Bazzano M, Piccione G, Giannetto C, Tosto F, Pietro SD, Giudice E. Platelet Rich Plasma Intralesional Injection as Bedside Therapy for Tendinitis in Athletic Horse. *Acta Sci Vet* 2013;**43**:1–7.

10. Beerts C, Suls M, Broeckx SY, Seys B, Vandenberghe A, Declercq J, et al. Tenogenically Induced Allogeneic Peripheral Blood Mesenchymal Stem Cells in Allogeneic Platelet-Rich Plasma: 2-Year Follow-up after Tendon or Ligament Treatment in Horses. *Front Vet Sci* 2017;**4**:158.

11. Behfar M. Adipose-derived stromal vascular fraction improves tendon healing in rabbits. *Chin J Traumatol* 2011;**14**:329–35.

12. Behfar M. Comparative Study on Functional Effects of Allotransplantation of Bone Marrow Stromal Cells and Adipose Derived. *Cell J* 2014;**16**:263–70.

13. Behfar M, Sarrafzadeh-Rezaei F, Hobbenaghi R, Delirezh N, Dalir-Naghadeh B. Enhanced Mechanical Properties of Rabbit Flexor Tendons in Response to Intratendinous Injection of Adipose Derived Stromal Vascular Fraction. *Curr Stem Cell Res Ther* 2012;**7**:173–8.

14. Bell R, Boniello MR, Gendron NR, Flatow EL, Andarawis-Puri N. Delayed exercise promotes remodeling in sub-rupture fatigue damaged tendons. *J Orthop Res* 2015;**33**:919–25.

15. Bell R, Gendron NR, Anderson M, Flatow EL, Andarawis-Puri N. A potential new role for myofibroblasts in remodeling of sub-rupture fatigue tendon injuries by exercise. *Sci Rep* 2018;**8**:8933–42.

16. Bring DK, Kreicbergs A, Renstrom PA, Ackermann PW. Physical activity modulates nerve plasticity and stimulates repair after achilles tendon rupture. *J Orthop Res* 2007;**25**:164–72.

17. Calandruccio JH, Cannon TA, Wodowski AJ, Stephens BF, Smith RA. A mechanical and histologic comparative study of the effect of saline, steroid, autologous blood, and platelet-rich plasma on collagenase-induced Achilles tendinopathy in a rat model. *Curr Orthop Pract* 2015;**26**:E7–12.

18. Canapp SO, Canapp DA, Ibrahim V, Carr BJ, Cox C, Barrett JG. The Use of Adipose-Derived Progenitor Cells and Platelet-Rich Plasma Combination for the Treatment of Supraspinatus Tendinopathy in 55 Dogs: A Retrospective Study. *Front Vet Sci* 2016;**3**:61.

19. Caniglia CJ, Schramme MC, Smith RK. The effect of intralesional injection of bone marrow derived mesenchymal stem cells and bone marrow supernatant on collagen fibril size in a surgical model of equine superficial digital flexor tendonitis. *Equine Vet J* 2012;**44**:587–93.

20. Chan BP, Fu S, Qin L, Lee K, Rolf CG, Chan K. Effects of basic fibroblast growth factor (bFGF) on early stages of tendon healing: A rat patellar tendon model. *Acta Orthop Scand* 2000;**71**:513–8.

21. Chen HS, Su YT, Chan TM, Su YJ, Syu WS, Harn HJ, et al. Human Adipose-Derived Stem Cells Accelerate the Restoration of Tensile Strength of Tendon and Alleviate the Progression of Rotator Cuff Injury in a Rat Model. *Cell Transplant* 2015;**24**:509–20.

22. Chen J, Yu Q, Wu B, Lin Z, Pavlos NJ, Xu J, et al. Autologous Tenocyte Therapy for Experimental Achilles Tendinopathy in a Rabbit Model. *Tissue Eng Part A* 2011;**17**:2037–48.

23. Chen L, Dong SW, Liu JP, Tao X, Tang KL, Xu JZ. Synergy of tendon stem cells and platelet-rich plasma in tendon healing. *J Orthop Res* 2012;**30**:991–7.

24. Chen L, Liu JP, Tang KL, Wang Q, Wang GD, Cai XH, et al. Tendon Derived Stem Cells Promote Platelet-Rich Plasma Healing in Collagenase-Induced Rat Achilles Tendinopathy. *Cell Physiol Biochem* 2014;**34**:2153–68.

25. Chiou GJ, Crowe C, McGoldrick R, Hui K, Pham H, Chang J. Optimization of an Injectable Tendon Hydrogel: The Effects of Platelet-Rich Plasma and Adipose-Derived Stem Cells on Tendon Healing *In Vivo*. *Tissue Eng Part A* 2015;**21**:1579–86.

26. Circi E. The impact of platelet-rich plasma (PRP) injection timing on the healing of Achilles tendon injuries in rats. *Acta Orthop Traumatol Turc* 2015;**50**:366–72.

27. Çınar BM. The effects of extracorporeal shock waves on carrageenan-induced Achilles tendinitis in rats: a biomechanical and histological analysis. *Acta Orthop Traumatol Turc* 2013;**47**:266–72.

28. Conze P, van Schie HT, Weeren R van, Staszyk C, Conrad S, Skutella T, et al. Effect of autologous adipose tissue-derived mesenchymal stem cells on neovascularization of artificial equine tendon lesions. *Regen Med* 2014;**9**:743–57.

29. Crovace A, Lacitignola L, Rossi G, Francioso E. Histological and Immunohistochemical Evaluation of Autologous Cultured Bone Marrow Mesenchymal Stem Cells and Bone Marrow Mononucleated Cells in Collagenase-Induced Tendinitis of Equine Superficial Digital Flexor Tendon. *Vet Med Int* 2010;**2010**:1–10.

30. Dahlgren LA, van der Meulen MCH, Bertram JEA, Starrak GS, Nixon AJ. Insulin-like growth factor-I improves cellular and molecular aspects of healing in a collagenase-induced model of flexor tendinitis. *J Orthop Res* 2002;**20**:910–9.

31. Dallaudière B, Lempicki M, Pesquer L, Louedec L, Preux PM, Meyer P, et al. Efficacy of intra-tendinous injection of platelet-rich plasma in treating tendinosis: comprehensive assessment of a rat model. *Eur Radiol* 2013;**23**:2830–7.

32. Dallaudière B, Lempicki M, Pesquer L, Louedec L, Preux PM, Meyer P, et al. Acceleration of tendon healing using US guided intratendinous injection of bevacizumab: First pre-clinical study on a murine model. *Eur J Radiol* 2013;**82**:e823–8.

33. Dallaudiere B, Louedec L, Lenet MPJ, Pesquer L, Blaise E, Perozziello A, et al. The molecular systemic and local effects of intra- tendinous injection of Platelet Rich Plasma in tendinosis: preliminary results on a rat model with ELISA method. *Muscles Ligaments Tendons J* 2015;**5**:99–105.

34. Dallaudiere B, Zurlinden O, Perozziello A, Deschamps L, Larbi A, Louedec L, et al. Combined intra-tendinous injection of Platelet Rich Plasma and bevacizumab accelerates and improves healing compared to Platelet Rich Plasma in tendinosis: comprehensive assessment on a rat model. *Muscles Ligaments Tendons J* 2014;**4**:351–6.

35. de Wit T, de Putter D, Tra WM, Rakhorst HA, van Osch GJ, Hovius SE, et al. Auto-crosslinked hyaluronic acid gel accelerates healing of rabbit flexor tendons in vivo. *J Orthop Res* 2009;**27**:408–15.

36. Majewski M, Heisterbach P, Jaquiéry C, Dürselen L, Todorov A, Martin I, et al. Improved tendon healing using bFGF, BMP-12 and TGFβ1 in a rat model. *Eur Cell Mater* 2018;**35**:318–34.

37. Genç E, Yüksel S, Çağlar A, Beytemur O, Güleç MA. Comparison on effects of platelet-rich plasma versus autologous conditioned serum on Achilles tendon healing in a rat model. *Acta Orthop Traumatol Turc* 2020;**54**:438–44.

38. Devana SK, Kelley BV, McBride OJ, Kabir N, Jensen AR, Park SJ, et al. Adipose-derived Human Perivascular Stem Cells May Improve Achilles Tendon Healing in Rats. *Clin Orthop Relat Res* 2018;**476**:2091–100.

39. Dietrich F, Duré GL, Klein CP, Bampi VF, Padoin V, Silva VD, et al. Platelet-Rich Fibrin Promotes an Accelerated Healing of Achilles Tendon When Compared to Platelet-Rich Plasma in Rat. *World J Plast Surg* 2015;**4**:101–9.

40. Dirks RC, Galley MR, Childress PJ, Fearon AM, Scott A, Koch LG, et al. Uphill running does not exacerbate collagenase-induced pathological changes in the Achilles tendon of rats selectively bred for high-capacity running. *Connect Tissue Res* 2013;**54**:386–93.

41. Durgam SS, Stewart AA, Sivaguru M, Wagoner Johnson AJ, Stewart MC. Tendon‐derived progenitor cells improve healing of collagenase‐induced flexor tendinitis. *J Orthop Res* 2016;**34**:2162–71.

42. Dyson SJ. Medical management of superficial digital flexor tendonitis: a comparative study in 219 horses (1992-2000). *Equine Vet J* 2010;**36**:415–9.

43. Estrada RJ, van Weeren R, van de Lest CHA, Boere J, Reyes M, Ionita J-C, et al. Effects of Autologous Conditioned Plasma® (ACP) on the healing of surgically induced core lesions in equine superficial digital flexor tendon. *PHK* 2014;**30**:633–42.

44. Facon-Poroszewska M, Kiełbowicz Z, Prządka P. Influence of Radial Pressure Wave Therapy (RPWT) on collagenase-induced Achilles tendinopathy treated with Platelet Rich Plasma and Autologous Adipose Derived Stem Cells. *Pol J Vet Sci* 2019;**22**:743–51.

45. Faisal T, Asjid R, Qamar K, Akhtar N, Moeed K, Hussain T. Effect of Autologous Platelet-rich Plasma on Appearance of Tenocytes at Injured Achilles Tendon Entheses in Rabbits. *J Coll Physicians Surg Pak* 2019;**29**:1029–33.

46. Fedato RA, Francisco JC, Sliva G, de Noronha L, Olandoski M, Faria Neto JR, et al. Stem Cells and Platelet-Rich Plasma Enhance the Healing Process of Tendinitis in Mice. *Stem Cells Int* 2019;**2019**:1–9.

47. Fernández-Sarmiento JA, Domínguez JM, Granados MM, Morgaz J, Navarrete R, Carrillo JM, et al. Histological Study of the Influence of Plasma Rich in Growth Factors (PRGF) on the Healing of Divided Achilles Tendons in Sheep. *J Bone Joint Surg Am* 2013;**95**:246–55.

48. Forslund C, Bylander B, Aspenberg P. Indomethacin and celecoxib improve tendon healing in rats. *Acta Orthop Scand* 2003;**74**:465–9.

49. Fukawa T, Yamaguchi S, Watanabe A, Sasho T, Akagi R, Muramatsu Y, et al. Quantitative Assessment of Tendon Healing by Using MR T2 Mapping in a Rabbit Achilles Tendon Transection Model Treated with Platelet-rich Plasma. *Radiology* 2015;**276**:748–55.

50. Geburek F, Gaus M, van Schie HTM, Rohn K, Stadler PM. Effect of intralesional platelet-rich plasma (PRP) treatment on clinical and ultrasonographic parameters in equine naturally occurring superficial digital flexor tendinopathies – a randomized prospective controlled clinical trial. *BMC Vet Res* 2016;**12**:191–206.

51. Geburek F, Lietzau M, Beineke A, Rohn K, Stadler PM. Effect of a single injection of autologous conditioned serum (ACS) on tendon healing in equine naturally occurring tendinopathies. *Stem Cell Res Ther* 2015;**6**:126–39.

52. Godbout C, Ang O, Frenette J. Early voluntary exercise does not promote healing in a rat model of Achilles tendon injury. *J Appl Physiol* 2006;**101**:1720–6.

53. Godwin EE, Young NJ, Dudhia J, Beamish IC, Smith RK. Implantation of bone marrow-derived mesenchymal stem cells demonstrates improved outcome in horses with overstrain injury of the superficial digital flexor tendon. *Equine Vet J* 2012;**44**:25–32.

54. González JC, López C, Álvarez ME, Pérez JE, Carmona JU. Autologous leukocyte-reduced platelet-rich plasma therapy for Achilles tendinopathy induced by collagenase in a rabbit model. *Sci Rep* 2016;**6**:19623.

55. Greimers L, Drion PV, Colige A, Libertiaux V, Denoël V, Lecut C, et al. Effects of Allogeneic Platelet-Rich Plasma (PRP) on the Healing Process of Sectioned Achilles Tendons of Rats: A Methodological Description. *J Vis Exp* 2018:55759.

56. Halici M, Karaoglu S, Canoz O, Kabak S, Baktir A. Sodium hyaluronate regulating angiogenesis during Achilles tendon healing. *Knee Surg Sports Traumatol Arthrosc* 2004;**12**:562–7.

57. Hammerman M, Dietrich-Zagonel F, Blomgran P, Eliasson P, Aspenberg P. Different mechanisms activated by mild versus strong loading in rat Achilles tendon healing. *PLoS ONE* 2018;**13**:e0201211.

58. Hankemeier S, Hurschler C, Zeichen J, van Griensven M, Miller B, Meller R, et al. Bone Marrow Stromal Cells in a Liquid Fibrin Matrix Improve the Healing Process of Patellar Tendon Window Defects. *Tissue Eng Part A* 2009;**15**:1019–30.

59. Ho LK, Baltzer WI, Nemanic S, Stieger-Vanegas SM. Single ultrasound-guided platelet-rich plasma injection for treatment of supraspinatus tendinopathy in dogs. *Can Vet J* 2015;**56**:845–9.

60. Hsu RW-W, Hsu W-H, Tai C-L, Lee K-F. Effect of shock-wave therapy on patellar tendinopathy in a rabbit model. *J Orthop Res* 2004;**22**:221–7.

61. Hunter J, McClure SR, Merritt DK, Reinertson E. Extracorporeal shockwave therapy for treatment of superficial digital flexor tendonitis in racing Thoroughbreds: 8 clinical cases. *Vet Comp Orthop Traumatol* 2004;**17**:152–5.

62. Jiang G, Wu Y, Meng J, Wu F, Li S, Lin M, et al. Comparison of Leukocyte-Rich Platelet-Rich Plasma and Leukocyte-Poor Platelet-Rich Plasma on Achilles Tendinopathy at an Early Stage in a Rabbit Model. *Am J Sports Med* 2020;**48**:1189–99.

63. Kaux JF, Janssen L, Drion P, Nusgens B, Libertiaux V, Pascon F, et al. Vascular Endothelial Growth Factor-111 (VEGF-111) and tendon healing: preliminary results in a rat model of tendon injury. *Muscle Ligaments and Tendons J* 2019;**04**:24–8.

64. Kaux JF, Drion PV, Colige A, Pascon F, Libertiaux V, Hoffmann A, et al. Effects of platelet-rich plasma (PRP) on the healing of Achilles tendons of rats: PRP and tendon healing. *Wound Repair Regen* 2012;**20**:748–56.

65. Kaux JF, Libertiaux V, Dupont L, Colige A, Denoël V, Lecut C, et al. Platelet-rich plasma (PRP) and tendon healing: comparison between fresh and frozen-thawed PRP. *Platelets* 2020;**31**:221–5.

66. Kaux JF, Libertiaux V, Leprince P, Fillet M, Denoel V, Wyss C, et al. Eccentric Training for Tendon Healing After Acute Lesion: A Rat Model. *Am J Sports Med* 2017;**45**:1440–6.

67. Kim MY, Farnebo S, Woon CYL, Schmitt T, Pham H, Chang J. Augmentation of Tendon Healing with an Injectable Tendon Hydrogel in a Rat Achilles Tendon Model. *Plast Reconstr Surg* 2014;**133**:645e–53e.

68. Kim SJ, Lee SM, Kim JE, Kim SH, Jung Y. Effect of platelet-rich plasma with self-assembled peptide on the rotator cuff tear model in rat: SAP-PRP effect on rotator cuff tear. *J Tissue Eng Regen Med* 2017;**11**:77–85.

69. Koenig J, Cruz A, Genovese R, Fretz P, Trostle S. Rupture of the peroneus tertius tendon in 27 horses. *Can Vet J* 2005;**46**:503–6.

70. Kraus TM, Imhoff FB, Reinert J, Wexel G, Wolf A, Hirsch D, et al. Stem cells and bFGF in tendon healing: Effects of lentiviral gene transfer and long-term follow-up in a rat Achilles tendon defect model. *BMC Musculoskelet Disord* 2016;**17**:148–54.

71. Kwon DR, Park GY, Lee SC. Treatment of Full-Thickness Rotator Cuff Tendon Tear Using Umbilical Cord Blood-Derived Mesenchymal Stem Cells and Polydeoxyribonucleotides in a Rabbit Model. *Stem Cells Int* 2018;**2018**:1–11.

72. Kwon DR, Park GY, Lee SC. Regenerative effects of mesenchymal stem cells by dosage in a chronic rotator cuff tendon tear in a rabbit model. *Regen Med* 2019;**14**:1001–12.

73. Kwon DR, Park GY, Moon YS, Lee SC. Therapeutic Effects of Umbilical Cord Blood-Derived Mesenchymal Stem Cells Combined with Polydeoxyribonucleotides on Full-Thickness Rotator Cuff Tendon Tear in a Rabbit Model. *Cell Transplant* 2018;**27**:1613–22.

74. Lacitignola L, Staffieri F, Rossi G, Francioso E, Crovace A. Survival of bone marrow mesenchymal stem cells labelled with red fluorescent protein in an ovine model of collagenase-induced tendinitis. *Vet Comp Orthop Traumatol* 2014;**27**:204–9.

75. Lamplot JD, Angeline M, Angeles J, Beederman M, Wagner E, Rastegar F, et al. Distinct Effects of Platelet-Rich Plasma and BMP13 on Rotator Cuff Tendon Injury Healing in a Rat Model. *Am J Sports Med* 2014;**42**:2877–87.

76. Lange-Consiglio A, Tassan S, Corradetti B, Meucci A, Perego R, Bizzaro D, et al. Investigating the efficacy of amnion-derived compared with bone marrow–derived mesenchymal stromal cells in equine tendon and ligament injuries. *Cytotherapy* 2013;**15**:1011–20.

77. Lee HJ, Kim YS, Ok JH, Lee YK, Ha MY. Effect of a single subacromial prednisolone injection in acute rotator cuff tears in a rat model. *Knee Surg Sports Traumatol Arthrosc* 2015;**23**:555–61.

78. Leeman JJ, Shaw KK, Mison MB, Perry JA, Carr A, Shultz R. Extracorporeal shockwave therapy and therapeutic exercise for supraspinatus and biceps tendinopathies in 29 dogs. *Vet Rec* 2016;**179**:385–385.

79. Li S, Wu Y, Jiang G, Tian X, Hong J, Chen S, et al. Intratendon delivery of leukocyte‐rich platelet‐rich plasma at early stage promotes tendon repair in a rabbit Achilles tendinopathy model. *J Tissue Eng Regen Med* 2020;**14**:452–63.

80. Liang JI, Lin PC, Chen MY, Hsieh TH, Chen JJ, Yeh ML. The effect of tenocyte/hyaluronic acid therapy on the early recovery of healing Achilles tendon in rats. *J Mater Sci Mater Med* 2014;**25**:217–27.

81. López-Nájera D, Rubio-Zaragoza M, Sopena-Juncosa JJ, Alentorn-Geli E, Cugat-Bertomeu R, Fernández-Sarmiento JA, et al. Effects of plasma rich in growth factors (PRGF) on biomechanical properties of Achilles tendon repair. *Knee Surg Sports Traumatol Arthrosc* 2016;**24**:3997–4004.

82. Lyras DN, Kazakos K, Georgiadis G, Mazis G, Middleton R, Richards S, et al. Does a Single Application of PRP Alter the Expression of IGF-I in the Early Phase of Tendon Healing? *J Foot Ankle Surg* 2011;**50**:276–82.

83. Ma R, Schär M, Chen T, Wang H, Wada S, Ju X, et al. Use of Human Placenta-Derived Cells in a Preclinical Model of Tendon Injury. *J Bone Joint Surg Am* 2019;**101**:e61.

84. Machova Urdzikova L, Sedlacek R, Suchy T, Amemori T, Ruzicka J, Lesny P, et al. Human multipotent mesenchymal stem cells improve healing after collagenase tendon injury in the rat. *BioMed Eng OnLine* 2014;**13**:42.

85. Maia L, de Souza MV, Ribeiro Júnior JI, de Oliveira AC, Alves GES, dos Anjos Benjamin L, et al. Platelet-Rich Plasma in the Treatment of Induced Tendinopathy in Horses: Histologic Evaluation. *J Equine Vet Sci* 2009;**29**:618–26.

86. Maman E, Yehuda C, Pritsch T, Morag G, Brosh T, Sharfman Z, et al. Detrimental Effect of Repeated and Single Subacromial Corticosteroid Injections on the Intact and Injured Rotator Cuff: A Biomechanical and Imaging Study in Rats. *Am J Sports Med* 2016;**44**:177–82.

87. Marfe G, Rotta G, De Martino L, Tafani M, Fiorito F, Di Stefano C, et al. A new clinical approach: Use of blood-derived stem cells (BDSCs) for superficial digital flexor tendon injuries in horses. *Life Sci* 2012;**90**:825–30.

88. Marycz K, Toker NY, Grzesiak J, Wrzeszcz K, Golonka P. The therapeutic effect of autogenic adipose derived stem cells combined with autogenic platelet rich plasma in tendons disorders hi horses in vitro and in vivo research. *J Anim Vet Adv* 2012;**11**:4324–31.

89. McDougall RA, Canapp SO, Canapp DA. Ultrasonographic Findings in 41 Dogs Treated with Bone Marrow Aspirate Concentrate and Platelet-Rich Plasma for a Supraspinatus Tendinopathy: A Retrospective Study. *Front Vet Sci* 2018;**5**:98.

90. McWhorter JW, Francis RS, Heckmann RA. Influence of local steroid injections on traumatized tendon properties. A biomechanical and histological study. *Am J Sports Med* 1991;**19**:435–9.

91. Mkumbuzi NS, Chinyanga HM, Wood W, Mudambo SKT, Gova M. Effects of different frequencies of loading on healing in partial rupture of the Achilles tendon in a rat model. *Cent Afr J Med* 2015;**61**:11–7.

92. Murrell GAC, Jang D, Deng XH, Hannafin JA, Warren RF. Effects of Exercise on Achilles Tendon Healing in a Rat Model. *Foot Ankle Int* 1998;**19**:598–603.

93. Najafbeygi A, Fatemi MJ, Lebaschi AH, Jaber S, Husseini SA, Niazi M. Effect of Basic Fibroblast Growth Factor on Achilles Tendon Healing in Rabbit. *World J Plast Surg* 2017;**6**:26–32.

94. Nakamura K, Kitaoka K, Tomita K. Effect of eccentric exercise on the healing process of injured patellar tendon in rats. *J Orthop Sci* 2008;**13**:371–8.

95. Naterstad IF, Rossi RP, Marcos RL, Parizzoto NA, Frigo L, Joensen J, et al. Comparison of Photobiomodulation and Anti-Inflammatory Drugs on Tissue Repair on Collagenase-Induced Achilles Tendon Inflammation in Rats. *Photomed Laser Surg* 2018;**36**:137–45.

96. Ng GY, Ng CO, See KN. Comparison of therapeutic ultrasound and exercises for augmenting tendon healing in rats. *Ultrasound Med Biol* 2004;**30**:1539–43.

97. Ng GY, Fung DT. The Combined Treatment Effects of Therapeutic Laser and Exercise on Tendon Repair. *Photomed Laser Surg* 2008;**26**:137–41.

98. Nixon AJ, Dahlgren LA, Haupt JL, Yeager AE, Ward DL. Effect of adipose-derived nucleated cell fractions on tendon repair in horses with collagenase-induced tendinitis. *Am J Vet Res* 2008;**69**:928–37.

99. Norelli JB, Plaza DP, Stal DN, Varghese AM, Liang H, Grande DA. Tenogenically differentiated adipose-derived stem cells are effective in Achilles tendon repair in vivo. *J Tissue Eng* 2018;**9**:1–20.

100. Nourissat G, Diop A, Maurel N, Salvat C, Dumont S, Pigenet A, et al. Mesenchymal Stem Cell Therapy Regenerates the Native Bone-Tendon Junction after Surgical Repair in a Degenerative Rat Model. Rannou FP, editor. *PLoS ONE* 2010;**5**:e12248.

101. Okamoto N, Kushida T, Oe K, Umeda M, Ikehara S, Iida H. Treating Achilles Tendon Rupture in Rats with Bone-Marrow-Cell Transplantation Therapy. *J Bone Joint Surg Am* 2010;**92**:2776–84.

102. Oliva F, Maffulli N, Gissi C, Veronesi F, Calciano L, Fini M, et al. Combined ascorbic acid and T3 produce better healing compared to bone marrow mesenchymal stem cells in an Achilles tendon injury rat model: a proof of concept study. *J Orthop Surg Res* 2019;**14**:54–63.

103. Oryan A, Moshiri A, Meimandi Parizi AH, Raayat Jahromi A. Repeated administration of exogenous Sodium-hyaluronate improved tendon healing in an in vivo transection model. *J Tissue Viability* 2012;**21**:88–102.

104. Oshita T, Tobita M, Tajima S, Mizuno H. Adipose-Derived Stem Cells Improve Collagenase-Induced Tendinopathy in a Rat Model. *Am J Sports Med* 2016;**44**:1983–9.

105. Palmes D, Spiegel HU, Schneider TO, Langer M, Stratmann U, Budny T, et al. Achilles tendon healing: Long-term biomechanical effects of postoperative mobilization and immobilization in a new mouse model. *J Orthop Res* 2002;**20**:939–46.

106. Parafioriti A, Armiraglio E, Del Bianco S, Tibalt E, Oliva F, Berardi AC. Single injection of platelet-rich plasma in a rat Achilles tendon tear model. *Muscles Ligaments Tendons J* 2011;**1**:41–7.

107. Park GY, Kwon DR, Lee SC. Regeneration of Full-Thickness Rotator Cuff Tendon Tear After Ultrasound-Guided Injection With Umbilical Cord Blood-Derived Mesenchymal Stem Cells in a Rabbit Model. *Stem Cells Transl Med* 2015;**4**:1344–51.

108. Peltz CD, Sarver JJ, Dourte LM, Würgler-Hauri CC, Williams GR, Soslowsky LJ. Exercise following a short immobilization period is detrimental to tendon properties and joint mechanics in a rat rotator cuff injury model. *J Orthop Res* 2010;**28**:841–5.

109. Philip J, Hackl F, Canseco JA, Kamel RA, Kiwanuka E, Diaz-Siso JR, et al. Amnion-Derived Multipotent Progenitor Cells Improve Achilles Tendon Repair in Rats. *Eplasty* 2013;**13**:225–34.

110. Qianman B, Jialihasi A, Asilehan B, Kubai A, Aibek R, Wupuer A, et al. Active exercise promotes Achilles tendon healing and is accompanied by the upregulation of collapsin response mediator protein-2 in rats. *Mol Med Rep* 2017;**16**:2355–60.

111. Rajabi H, Shahin HS, Norouzian M, Mehrabani D, Nazhvani SD. The Healing Effects of Aquatic Activities and Allogenic Injection of Platelet-Rich Plasma (PRP) on Injuries of Achilles Tendon in Experimental Rat. *World J Plast Surg* 2015;**4**:66–73.

112. Renzi S, Riccò S, Dotti S, Sesso L, Grolli S, Cornali M, et al. Autologous bone marrow mesenchymal stromal cells for regeneration of injured equine ligaments and tendons: A clinical report. *Res Vet Sci* 2013;**95**:272–7.

113. Rezvani SN, Chen J, Li J, Midura R, Cali V, Sandy JD, et al. In‐Vivo Efficacy of Recombinant Human Hyaluronidase (rHuPH20) Injection for Accelerated Healing of Murine Retrocalcaneal Bursitis and Tendinopathy. *J Orthop Res* 2020;**38**:59–69.

114. Sarıkaya B, Yumuşak N, Yigin A, Sipahioğlu S, Yavuz Ü, Altay MA. Comparison of the effects of human recombinant epidermal growth factor and platelet-rich plasma on healing of rabbit patellar tendon. *Eklem Hastalik Cerrahisi* 2017;**28**:92–9.

115. See EK, Ng GY, Ng CO, Fung DT. Running exercises improve the strength of a partially ruptured Achilles tendon. *Br J Sports Med* 2004;**38**:597–600.

116. Sen B, Guler S, Cecen B, Kumtepe E, Bagriyanik A, Ozkal S, et al. The Effect of Autologous Platelet Rich Plasma in the Treatment of Achilles Tendon Ruptures: An Experimental Study on Rabbits. *Balkan Med J* 2016;**33**:94–101.

117. Shah V, Bendele A, Dines JS, Kestler HK, Hollinger JO, Chahine NO, et al. Dose-response effect of an intra-tendon application of recombinant human platelet-derived growth factor-BB (rhPDGF-BB) in a rat Achilles tendinopathy model. *J Orthop Res* 2013;**31**:413–20.

118. Shokry M, Mostafa A, Tohamy A, El-Sharkawi M. Autologous mesenchymal stem cells for treatment of acute superficial digital flexor tendonitis in athletic horses: clinical study of 15 cases. *PHK* 2020;**36**:43–8.

119. Smith RKW, Werling NJ, Dakin SG, Alam R, Goodship AE, Dudhia J. Beneficial Effects of Autologous Bone Marrow-Derived Mesenchymal Stem Cells in Naturally Occurring Tendinopathy. *PLoS ONE* 2013;**8**:e75697.

120. Solchaga LA, Bendele A, Shah V, Snel LB, Kestler HK, Dines JS, et al. Comparison of the effect of intra-tendon applications of recombinant human platelet-derived growth factor-BB, platelet-rich plasma, steroids in a rat achilles tendon collagenase model. *J Orthop Res* 2014;**32**:145–50.

121. Souza M, Moraes SAS, de Paula DR, Maciel AA, Batista EJO, Silva DGF, et al. Local treatment with ascorbic acid accelerates recovery of post-sutured Achilles tendon in male Wistar rats. *Braz J Med Biol Res* 2019;**52**:e8290.

122. Souza MV de, Moreira J do CL, Silva MO da, Crepaldi J, Silva CHO, Garcia SLR, et al. Histomorphometric analysis of the Achilles tendon of Wistar rats treated with laser therapy and eccentric exercise. *Pesq Vet Bras* 2015;**35**:39–50.

123. Depuydt E, Broeckx SY, Van Hecke L, Chiers K, Van Brantegem L, van Schie H, et al. The Evaluation of Equine Allogeneic Tenogenic Primed Mesenchymal Stem Cells in a Surgically Induced Superficial Digital Flexor Tendon Lesion Model. *Front Vet Sci* 2021;**8**:15.

124. Spang JT, Tischer T, Salzmann GM, Winkler T, Burgkart R, Wexel G, et al. Platelet concentrate vs. saline in a rat patellar tendon healing model. *Knee Surg Sports Traumatol Arthrosc* 2011;**19**:495–502.

125. Spurlock SL, Spurlock GH, Bernstad S, Michanek P, Chester ST. Treatment of acute superficial flexor tendon injuries in performance horses with high molecular weight sodium hyaluronate. *J Equine Vet Sci* 1999;**19**:338–44.

126. Tatari H, Skiak E, Destan H, Ulukuş Ç, Özer E, Satoğlu S. Effect of hylan G-F 20 in achilles’ tendonitis: An experimental study in rats. *Arch Phys Med Rehabil* 2004;**85**:1470–4.

127. Torricelli P, Fini M, Filardo G, Tschon M, Pischedda M, Pacorini A, et al. Regenerative medicine for the treatment of musculoskeletal overuse injuries in competition horses. *Int Orthop* 2011;**35**:1569–76.

128. Tosun HB, Gumustas SA, Kom M, Uludag A, Serbest S, Eroksuz Y. The Effect of Sodium Hyaluronate plus Sodium Chondroitin Sulfate Solution on Peritendinous Adhesion and Tendon Healing: An Experimental Study. *Balkan Med J* 2016;**33**:258–66.

129. Uzun C, Erdal N, Gürgül S, Kalaycı D, Yılmaz ŞN, Özdemir AA, et al. Comparison of the Effects of Pulsed Electromagnetic Field and Extracorporeal Shockwave Therapy in a Rabbit Model of Experimentally Induced Achilles Tendon Injury. *Bioelectromagnetics* 2021;**42**:128–45.

130. Virchenko O, Aspenberg P, Lindahl TL. Low molecular weight heparin impairs tendon repair. *J Bone Joint Surg Br* 2008;**90-B**:388–92.

131. Virchenko O, Aspenberg P. How can one platelet injection after tendon injury lead to a stronger tendon after 4 weeks? Interplay between early regeneration and mechanical stimulation. *Acta Orthopaedica* 2006;**77**:806–12.

132. Virchenko O, Grenegård M, Aspenberg P. Independent and additive stimulation of tendon repair by thrombin and platelets. *Acta Orthop* 2006;**77**:960–6.

133. Watts AE, Yeager AE, Kopyov OV, Nixon AJ. Fetal derived embryonic-like stem cells improve healing in a large animal flexor tendonitis model. *Stem Cell Res Ther* 2011;**2**:4–15.

134. Wei AS, Callaci JJ, Juknelis D, Marra G, Tonino P, Freedman KB, et al. The Effect of Corticosteroid on Collagen Expression in Injured Rotator Cuff Tendon. *J Bone Joint Surg Am* 2006;**88**:1331–8.

135. Williams IF, Nicholls JS, Goodship AE, Silver IA. Experimental treatment of tendon injury with heparin. *Br J Plast Surg* 1986;**39**:367–72.

136. Witte S, Dedman C, Harriss F, Kelly G, Chang Y-M, Witte TH. Comparison of treatment outcomes for superficial digital flexor tendonitis in National Hunt racehorses. *Vet J* 2016;**216**:157–63.

137. Yamaguchi T, Ochiai N, Sasaki Y, Kijima T, Hashimoto E, Sasaki Y, et al. Efficacy of hyaluronic acid or steroid injections for the treatment of a rat model of rotator cuff injury. *J Orthop Res* 2015;**33**:1861–7.

138. Yamamoto E, Hata D, Kobayashi A, Ueda H, Tangkawattana P, Oikawa M, et al. Effect of Beta-aminopropionitrile and Hyaluronic Acid on Repair of Collagenase-induced Injury of the Rabbit Achilles Tendon. *J Comp Pathol* 2002;**126**:161–70.

139. Yan R, Gu Y, Ran J, Hu Y, Zheng Z, Zeng M, et al. Intratendon Delivery of Leukocyte-Poor Platelet-Rich Plasma Improves Healing Compared With Leukocyte-Rich Platelet-Rich Plasma in a Rabbit Achilles Tendinopathy Model. *Am J Sports Med* 2017;**45**:1909–20.

140. Yang X, Meng H, Peng J, Xu L, wangW Y, Sun X, et al. Construction of Microunits by Adipose-Derived Mesenchymal Stem Cells Laden with Porous Microcryogels for Repairing an Acute Achilles Tendon Rupture in a Rat Model. *Int J Nanomedicine* 2020;**15**:7155–71.

141. Yea JH, Kim I, Sym G, Park JK, Lee AY, Cho BC, et al. Regeneration of a full-thickness defect in rotator cuff tendon with umbilical cord-derived mesenchymal stem cells in a rat model. *PLoS ONE* 2020;**15**:e0235239.

142. Yoo SD, Choi S, Lee GJ, Chon J, Jeong YS, Park HK, et al. Effects of extracorporeal shockwave therapy on nanostructural and biomechanical responses in the collagenase-induced Achilles tendinitis animal model. *Lasers Med Sci* 2012;**27**:1195–204.

143. Yoshida M, Funasaki H, Kubota M, Marumo K. Therapeutic effects of high molecular weight hyaluronan injections for tendinopathy in a rat model. *J Orthop Sci* 2015;**20**:186–95.

144. Yoshida M, Funasaki H, Marumo K. Efficacy of autologous leukocyte-reduced platelet-rich plasma therapy for patellar tendinopathy in a rat treadmill model. *Muscles Ligaments Tendons J* 2016;**6**:205–15.

145. Yüksel S, Adanır O, Gültekin MZ, Çağlar A, Küçükyıldırım BO, Güleç MA, et al. Effect of platelet-rich plasma for treatment of Achilles tendons in free-moving rats after surgical incision and treatment. *Acta Orthop Traumatol Turc* 2015;**49**:544–51.

146. Yuksel S, Guleç MA, Gultekin MZ, Adanır O, Caglar A, Beytemur O, et al. Comparison of the early period effects of bone marrow-derived mesenchymal stem cells and platelet-rich plasma on the Achilles tendon ruptures in rats. *Connect Tissue Res* 2016;**57**:360–73.

147. Zhang F, Liu H, Stile F, Lei M-P, Pang Y, Oswald TM, et al. Effect of Vascular Endothelial Growth Factor on Rat Achilles Tendon Healing. *Plast Reconstr Surg* 2003;**112**:1613–9.

148. Zhang J, Yuan T, Zheng N, Zhou Y, Hogan MV, Wang JH. The combined use of kartogenin and platelet-rich plasma promotes fibrocartilage formation in the wounded rat Achilles tendon entheses. *Bone Joint Res* 2017;**6**:231–44.

149. Zhang J, Middleton KK, Fu FH, Im HJ, Wang JH. HGF Mediates the Anti-inflammatory Effects of PRP on Injured Tendons. *PLoS ONE* 2013;**8**:e67303.

150. Uzun C, Erdal N, Gürgül S, Kalaycı D, Yılmaz ŞN, Özdemir AA, et al. Comparison of the Effects of Pulsed Electromagnetic Field and Extracorporeal Shockwave Therapy in a Rabbit Model of Experimentally Induced Achilles Tendon Injury. *Bioelectromagnetics* 2021;**42**:128-45.
